# A Systematic Review and Provisional Metanalysis on Psychopathologic Burden on Health Care Workers of Coronavirus Outbreaks

**DOI:** 10.3389/fpsyt.2020.568664

**Published:** 2020-10-16

**Authors:** Federica Galli, Gino Pozzi, Fabiana Ruggiero, Francesca Mameli, Marco Cavicchioli, Sergio Barbieri, Maria Paola Canevini, Alberto Priori, Gabriella Pravettoni, Gabriele Sani, Roberta Ferrucci

**Affiliations:** ^1^European Institute of Oncology, Milan, Italy; ^2^Asst SS.Paolo e Carlo, S.Paolo Hospital, Milan, Italy; ^3^Department of Neuroscience, Section of Psychiatry, Università Cattolica del Sacro Cuore, Rome, Italy; ^4^Department of Psychiatry, Fondazione Policlinico Universitario Agostino Gemelli IRCCS, Rome, Italy; ^5^Fondazione IRCCS ca’ Granda Ospedale Maggiore Policlinico, Milan, Italy; ^6^Unit of Clinical Psychology and Psychotherapy, Department of Psychology, University “Vita-Salute San Raffaele”, San Raffaele-Turro Hospital, Milan, Italy; ^7^Department of Health Science, University of Milan, Milan, Italy; ^8^Aldo Ravelli Center, Department of Health Science, University of Milan, Milan, Italy; ^9^Department of Oncology and Hemato-Oncology, University of Milan, Milan, Italy

**Keywords:** Post-Traumatic Stress Disorder, anxiety, depression, psychological distress, “health care worker”

## Abstract

**Background:**

The new coronavirus (SARS-CoV-2) shows several similarities with previous outbreaks of Severe Acute Respiratory Syndrome (SARS) and Middle East Respiratory Syndrome (MERS). Aim of this systematic review and meta-analysis is to provide evidence of the psychopathologic burden on health care workers (HCWs) of the first two deadly coronavirus outbreaks to get lessons for managing the current burden of COVID-19 outbreak.

**Method:**

According to Cochrane Collaboration guidelines and the PRISMA Statement, the study quantified the effects of frontline work on mental health of HCWs. Major databases — Pubmed, Scopus, Embase, Medline, and Web of Science — were searched for observational and case-control studies evaluating mental health indexes reported by front-line work. This study computed the percentage of sample that reported clinically significant levels of psychiatric symptoms. Cohen’s *d* was used for comparing mental health outcomes of health care workers directly involved in addressing pandemic emergency with a control group that was not directly exposed to such conditions. Pooled effect sizes (*d_w_*) were estimated whenever at least three independent studies yielded data. Heterogeneity of findings and bias of publication were estimated as well.

**Findings:**

Fifteen studies have been selected for a total of 7,393 HCWs. From 9.6% to 51% of HCWs reported symptoms of Post-Traumatic Stress Disorder (PTSD) and from 20% to 75% reported psychiatric symptoms, with a prevalence of anxiety and depression. From one to the three years after outbreak, from 2% to 19% reported PTSD symptoms and from 5% to 90% psychiatric symptoms. Interestingly, HWCs who were directly involved in pandemic emergency showed significantly higher depressive and anxious symptoms (*d_w_* = .66 (.46–.85); p <.001) than ones who were not directly exposed. Similarly, the direct involvement significantly affected the severity of PTSD symptoms (*d_w_* = .30 (.21–.39); p <.001).

**Conclusion:**

Health care professionals in general and most of all frontline workers showed an association with a likely risk of developing psychiatric disorders following outbreaks and for at least three years later. Mental health interventions for professionals exposed to COVID-19 need to be immediately implemented. Further studies are warranted to investigate long-term consequences carefully, and to look for mediating and buffering factors as well. The role of clinical psychologists and psychiatrists in delivering adequate interventions is critically important.

## Introduction

Several viral diseases have emerged and impacted healthcare systems worldwide. Apart from the pure medical response, a major issue in dealing with viral pandemic is the human aspect.

The novel coronavirus infection (SARS-CoV-2) and related syndrome (COVID-19) was first identified in Wuhan, China, in December 2019 (1), with a declaration of pandemic on March 11, 2020 (2). Previous coronavirus outbreaks resulted in a major global public crisis. In November 2002, in China’s Guangdong, Severe Acute Respiratory Syndrome (SARS-CoV) was first detected. It lasted 80 days (from mid-March 2003 till 31 May 2003) when Singapore was removed from the World Health Organization (WHO) list of SARS (3). SARS was characterized by atypical pneumonia and droplet transmission.

The SARS outbreak had an important concentration in health care settings and a large number of health care workers who have been infected, with an estimate of more than 20% of those who contracted the disease (3). During the SARS outbreak, more than 8,000 individuals in 29 countries were infected over 7 months (4).

After the emergence of SARS, the Middle East Respiratory Syndrome (MERS-CoV) was the second coronavirus infection resulting in a major global public health crisis. It first emerged in 2012 in Saudi Arabia (5, 6), with an outbreak infection occurring in Korea from May to December 2015. The virus caused a total of 2,279 cases from 27 countries, till the end of February 2019 (7), with health care workers who continue to be at higher risk of being affected (1).

The COVID-19 showed several similarities with the SARS, and MERS, about the clinical presentations, which can vary from asymptomatic infection to severe or fatal disease and it is highly transmissible. The most common onset symptoms of the COVID-19 include fever, dry cough, muscle pains, lethargy and fatigue. However, the spread of COVID-19 infection is much broader than SARS or MERS and involves larger numbers of patients (8). From now, COVID-19 killed a higher number of people than MERS and SARS together, in spite of a fatality rate around 2%, compared to a case fatality rate of around 10% for SARS, with 34% of affected people killed by MERS between 2012 and 2019 (9).

All the physicians and nurses embedded in emergency care are under extreme psychological pressure and are at high risk of developing psychological diseases, with protracted working hours and unexpected changes in the sort of work (10). This situation may result in severe psychological distress and could lead to burnout (11). The analysis of the psychopathologic burden of previous outbreaks may help to understand the likely consequences for HCWs of the current pandemic of COVID-19, to plan psychological interventions and prevent future negative outcomes.

The objective of our study is to provide a systematic review of the psychological and psychopathological burden on HCWs of the two first deadly coronavirus outbreaks (SARS and MERS).

## Methods

The objective of this systematic review is to analyze all observational studies realized on the burden on mental health of caring for patients affected by MERS and SARS. The case-control study design, adequacy of sample size, comparison and outcome measures have been all carefully analyzed to guarantee the right inclusion of selected studies.

### Search Strategy

Electronic searches were conducted on the major databases in the field of health and social sciences — Pubmed, Scopus, Embase, Medline, and Web of Science — in order to include the broadest range of relevant literature.

The selection of the search terms is based on the clinical experience and the topic literature on mental health (12). The search was performed using Mesh terms/Keywords (depending on the database) with the same search strategy: “Health Worker” AND “Epidemic” OR “MERS” OR “SARS” OR “Outbreak” AND “Depression” OR “Anxiety” OR “Burnout” OR “PTSD” OR “Suicide”.

**Figure 1:**
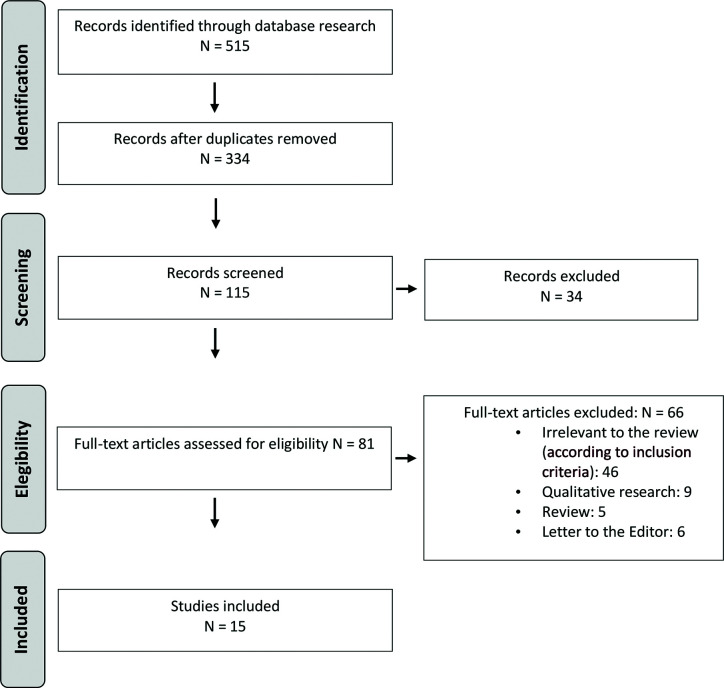
PRISMA flow diagram of literature search and selection of publications.

The search was limited to English-written publications, and to the period from 2002 to April 2020. When the full text was not retrievable, the study was excluded. Study selection was performed by independent reviewers with research expertise in clinical psychology who assessed the relevance of the study for the objectives of this review (**Figure 1**).

An additional analysis of the reference list was performed in each selected paper as well. When the full text was not retrievable, the study was excluded. It has been selected a final number of fifteen studies.

Inclusion Criteria:

- Studies should report data on mental health indexes linked to epidemic infections (SARS, MERS).
- Studies with an analytical study design as defined by Grimes and Schulz (13) (i.e., an observational study with a comparison or control group).
- Studies adopting standardized and validated instruments to assess psychological factors.
- Studies written in English.

Exclusion Criteria:

- Case reports, reviews, Letters to the Editor.
- Number of subjects per group ≤5.
- Qualitative studies.

### Data Extraction

Study selection was performed by independent reviewers with research expertise in clinical psychology (FG, FM, RF) who assessed the relevance of the study for the objectives of this review. This first round of selection was based on the title, abstract, and keywords of each study. If the reviewers did not reach a consensus or the abstract did not contain sufficient information, the full text was reviewed.

In the second phase (screening), full-text reports have been evaluated to detect whether the studies met the inclusion criteria (**Figure 1**). In the phase of eligibility, full texts have been retrieved, and a final check was made to exclude papers not responding to inclusion/exclusion criteria, and reaching the final consensus to decide the final number of studies to be selected.

A standardized data extraction form was prepared; data was independently extracted by two of the authors (FG and RF) and inserted in a study database (Cohen’s *k = *.85) (14).

A process of discussion/consensus moderated by a third reviewer (GP) (15) resolved discrepancies between reviewers (for three studies).

### Statistical Methods

**Figure 2:**
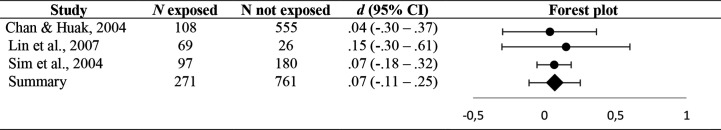
Forest plot of overall psychiatric symptoms.

**Figure 3:**
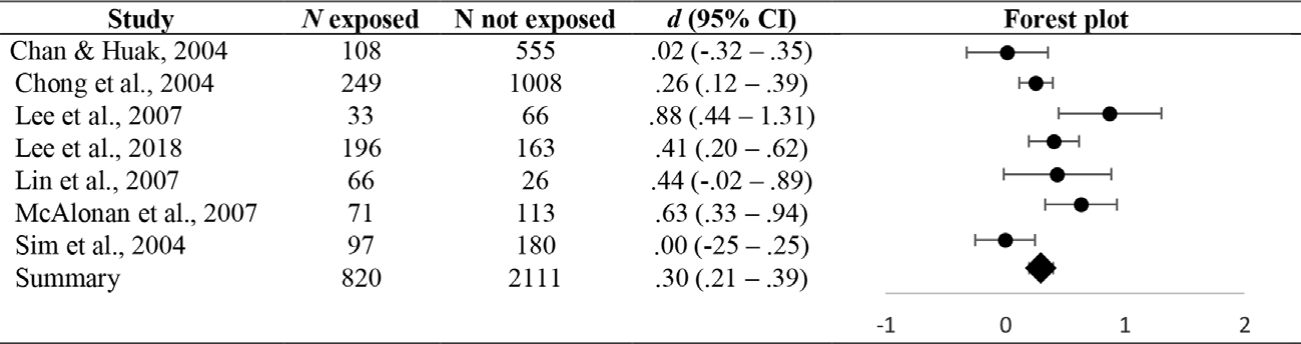
Forest plot of PTSD symptoms.

**Figure 4:**
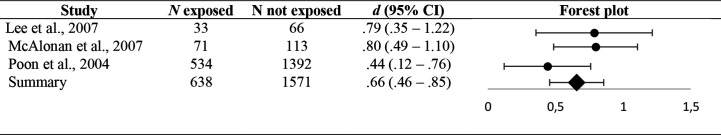
Forest plot of depression and anxious symptoms.

A systematic analysis was conducted according to the Cochrane Collaboration guidelines (15) and the PRISMA Statement (16). The current review provided a quantitative approach for aggregating results of studies considering as the main outcomes the percentage of sample that reported clinically significant levels of overall and specific psychiatric symptoms (i.e., PTSD, depression and anxiety) (**Figures 2**–**4**) (for a description of cut-off scores see **Table 1**). Furthermore, this work aims at quantifying mental health consequences of the direct exposure to clinical management of pandemic emergency. Accordingly, meta-analytic procedures were conducted comparing levels of different mental health outcomes of health care workers directly involved in addressing pandemic emergency to a control group that was not directly exposed to such conditions. Cohen’s *d* (32) was used as measure of effect size. Cohen’s *d* was primarily calculated using descriptive statistics reported in the Results section of each study. Values of Cohen’s *d* less than or equal to.20,.50, and.80 were interpreted as small, moderate, and large effect sizes, respectively (32). The overall pooled effect sizes (*dw*) for each mental health outcomes were estimated using the weighted mean of *d* value for each study (33, 34). The 95% confidence interval (CI) was computed, as was its significance according to the ratio of pooled effect size to the standard error (33, 34). Pooled effect sizes were estimated whenever at least three independent studies yielded data. Heterogeneity in effect sizes was computed using the *Q* statistic (34) and *I*^2^ index (14, 35). Excel was used to compute these metrics.

**Table 1:** Overview of selected studies.

Study	Sample description	Country	Disease	Study design	Timing	Assessment tools	Outcome measure	% of clinical distress	Effect size(95% CI)	Other significant findings
(17)	N=661(113 doctors; 548 nurses)	Singapore	SARS	Cross-sectional survey Case-control study: Direct exposure vs nondirect exposure	2-months after first case	GHQ-28(cut-off > 5)IES(cut-off >30)	Psychiatric symptoms PTSD symptoms	Psychiatric symptoms 27% (Doctors: 35%; Nurses: 25%) PTSD 19.2% (Doctors: 19.4%; Nurses: 19.3%)	Psychiatric symptoms Doctors: *d* = .14 (−.25–.53) Nurses: *d* = −.06 (−.29–.17) PTSD symptoms Doctors: *d* = −.02 (−.43–.39) Nurses: *d* = .05 (−.18–.28)	Clear communication of directives/precautionary measures (p=.020) and support from supervisors/colleagues (p=.003) are protective factors. No difference between doctors and nurses. No significant difference between those who were or were not exposed to SARS patients
(18)	N=1,257(676 nurses;139 doctors;140 health administrative workers; others health professionals)	Taiwan	SARS	Cross-sectional survey Case-control study Direct exposure vs nondirect exposure	6 weeks (during serious nosocomial infection)	Chinese Health Questionnaire(cut-off > 2) IES (cut-off not reported)	Psychiatric morbidity PTSD symptoms	75.3% psychiatric comorbidity	PTSD symptoms*d* = .26 (.12–.40)	-Differences between initial phase and second phase
(19)	N=271 HCWs; N=342 HCs	Hong Kong	SARS	Case-control study HCWs vs HC	During outbreak	PSS (cut-off not reported)	perceived stress	Not reported	Not available data	HCWs were not more stressed than healthy control subjects
(20)	N=139 (74% nurses; 15% employees; 11% clerical staff)	Toronto, Hamilton (Ontario)	SARS	Follow-up study	-one/two years after outbreak	SCID CAPS	Psychiatric disorders	5% any new onset of a psychiatric disorder 4% new episodes of Major Depression 2% new PTSD	Not available data	Any axis I diagnosis correlates with a previous psychiatric history (p=.02)(protective) association with years of health care experience (p=.03) and perception of hospital support and training (p=.03)
(21)	N=99 (63 nonhealth care workers vs 33 health care workers survivors to outbreak)	Hong Kong	SARS	Case-control HCWs vs HCs	−1 year after outbreak	GHQ-12 (cut-off > 3) PSS-10 IES-R DASS-21	Psychiatric morbidity Psychological distress PTSD symptoms Depressive and anxiety symptoms	Overall psychiatric morbidity 64% Health care workers 90.3% Nonhealth care workers 49.1%	Psychological distress *d* = .44 (.03–.85) PTSD symptoms *d* = .88 (.45–1.31) Depressive symptoms *d* = .70 (.27–1.13) Anxiety symptoms *d* = .87 (.44–1.30)	Health care workers:>depression(p<.01), >anxiety (p=.001), >PTSD symptoms (p=.05) -77.4% of female SARS survivors scored above the GHQ-12 threshold
(22)	N= 359 HCW (196 nurses, 30 doctors, 55 medical technicians, 31 administrators, 8 pharmacists; 39 others)	South Korea	MERS	Cross-sectional survey and case-control study Direct exposure vs nondirect exposure	During outbreak and one month after	IES-R (cut-off > 25)	PTSD symptoms	51%	PTSD symptoms *d* = .40 (.20–.60)	Trend differences between nurses and doctors (p=.048)
(23)	N=92 (66 HCW in emergency department vs 26 HCW in psychiatric ward)	Taichung (Taiwan)	SARS	Case-control	-one-month after outbreak	CHQ-12 (cut-off > 3) Davidson Trauma Scale-Chinese version (cut-off > 40)	Psychiatric comorbidity PTSD symptoms	Overall psychiatric morbidity 47.7% PTSD symptoms 19.3%	Psychological distress *d* = .15 (−.29–.59) PTSD symptoms *d* = .44 (.00–.88)	-HCW of ED showed more PTSD symptoms than HCW of psychiatric ward (p<.05) -No difference in CHQ - 93% of medical staff considered the SARS outbreak as a traumatic experience.
(24)	*N*=549 hospital employees	Beijing	SARS	Cross-sectional survey	-3 years after outbreak	CES-D (cut-off > 25)	Depressive symptoms	Depressive symptoms 8.8%	Not available data	-having been quarantined (p<.001), high work exposure (p<.001), current stressful job (p<.001), high PTSD symptoms (p<.001) and pre-SARS trauma exposure (p<.01) significantly predicted high depressive symptoms.-Altruistic acceptance of SARS-related risk was negatively associated (p=.0005)
(25)	N=769 (73.5% nurses, 8.3% clerical staff, 2.9% doctors, 2.3% respiratory therapists)	Toronto, Hamilton (Ontario)	SARS	Cross-sectional survey Case-control Toronto Vs Hamilton	-19 months after outbreak	K10 (cut-off > 16) Maslach Burnout Inventory (cut-off > 27) IES (cut-off > 26)	Psychological distress Burnout PTSD symptoms	Psychological distress 37.5% Burnout 24.8% PTSD symptoms 11.1%	Psychological distress *d* = .34 (.13–.55) Burnout *d* = .33 (.12–.54) PTSD symptoms *d* = .31 (.00–.62)	Maladaptive coping and perceived adequacy of training with protection and support explained 18% of the variance in burnout. - Maladaptive coping and attachment anxiety, together with a protective effect of experience in healthcare, explained 31% of the variance in psychological distress.
(26)	N=184 (71 high-risk HCW and 113 low-risk) (2004)	Hong Kong	SARS	Case-control study	-during (2003) and one year (2004) after outbreak	PSS-10 DASS-21 IES-R	Psychological distress Depressive anxious symptoms PTSD symptoms	Not reported	Psychological distress *d* = .76 (.47–1.03) Depressive symptoms *d* = .75 (.26– 1.02) Anxiety symptoms *d* = .84 (.55–1.13) PTSD symptoms *d* = .63 (.34–.92)	-in 2003, equally high perceived stress levels (p=.176) -in 2004, perceived stress decreased only in low risk HCW (p<.05) -in 2004, no differences in perceived stress among doctors, nurses, and others -PTSD symptoms correlated with exposure to SARS (p<.001)
(27)	N=510	Toronto	SARS	Cross-sectional survey	-during outbreak	GHQ-12 (cut-off > 3)	Psychiatric symptoms	29%	Not available data	-45.1% nurses, 33.3% allied health care professionals, 17.4% doctors, 18.9% staff not working in patient care
(28)	N=1926 (813 nurses; 141 doctors; 349 supporting staff; 230 administrative staff; 207 allied health workers; 186 others)	Hong Kong	SARS	Case-control Front-line health care workers vs Administrative Controls Contact with SARS Vs No contact with SARS	-two months during outbreak	STAI Maslach Burnout Inventory	Anxiety Burnout score	Not reported	Anxiety symptoms *d* = .41 (−0.2–.84) *d* = .47 (.37–.57) Burnout *d* = .61 (.19–1.03) *d* = .47 (.37–.57)	- Anxiety was higher among front-line HCW than administrative staff controls (p<.001). - Anxiety scores correlated (p<.001) with burnout scores among front-line HCW (*r*=0.58), controls (*r*=0.52), staff with contact with SARS patients (*r*=0.59), and staff without contact (*r*=0.56).
(29)	N=277 (91 doctors and 186 nurses)	Singapore	SARS	Cross-sectional survey Case-control study Direct exposure vs indirect exposure	4 months after outbreak	GHQ-28 (cut-off > 5) IES-R (cut-off > 3)	Psychiatric morbidity PTSD symptoms -	Psychiatric morbidity 20.6% PTSD symptoms 9.6%	Psychological distress *d* = .07 (−.18–.32) PTSD symptoms *d* = .00 (−.25–.25)	-No differences between doctors and nurses in the outcome measures
(30)	N=124 (41 doctors and 83 nurses)	Singapore	SARS	Cross-sectional survey	-6 months after outbreak	GHQ-28 (cut-off > 5) IES (cut-off >26)	Psychiatric morbidity PTSD symptoms	Psychiatric morbidity 18.8% PTSD symptoms 17.7%	Not available data	- Nurses reported higher morbidity rates
(31)	N=549 hospital employees	Beijing	SARS	Cross-sectional survey	3 years after outbreak	IES-R (cut-off >20)	PTSD symptoms	PTSD symptoms 10%	Not available data	-40% of PTSD symptoms continue to show symptoms after three years - altruism correlate with low PTSD

CAPS, Clinician-Administered PTSD Scale; CES-D, Center for Epidemiologic Studies Depression Scale; CHQ-12, Chinese Health Questionnaire-12; DASS-21, 21-item Depression Anxiety Stress Scales; ED, Emergency Department; GHQ, General Health Questionnaire; HADS, Hospital Anxiety and Depression Scale; HCW, Health Care Workers; IES, Impact of Events Scale; MERS, Middle East respiratory syndrome; MINI, Mini International Neuropsychiatric Interview; K10, Kessler Psychological Distress Scale; PSS-10, 10-item Perceived Stress Scale; SARS, Severe Acute Respiratory Syndrome; SCID, Structured Clinical Interview for DSM-IV; STAI, State-Trait Anxiety.

Despite the small number of studies for each outcome, Egger’s regression (i.e., the standard normal deviate [SND] is regressed against the estimate’s precision, defined as the inverse of the standard error; SND = a + *b* × precision) (36) was performed to detect publication bias. These analyses were conducted using SPSS 22.

### Risk of Bias

The current systematic review assessed quality of studies included using the rating scale developed by the National Institutes of Health for observational cohort and cross-sectional research designs (37). This scale is composed of 14 items rated on three levels (i.e., Yes; No; Cannot determine/Not applicable/Not reported [CD, NA, NR]) where a “no” or “undetermined” response indicates the presence of possible bias. The quality of each study was independently assessed by two authors (GP and FG), who reached a high inter-rater reliability (Cohen’s k = .89). At the end of the evaluation, ratings of each study were summed up within each item in order to provide a quantitative approach to the assessment of risk of bias. Given the number of studies included in this review, the total score (i.e., 210) was divided in three subscales capturing strengths (i.e., Yes responses), biases (No responses) and qualities not applicable (NA response). For a detailed description of results of these procedures, see **Table 4**.

## Results

A total of 7,393 HCWs has been scrutinized by the all studies (**Table 1**). Descriptive analysis of the all studies are reported in **Table 2**. Data are drawn from survey with voluntary and anonymous participation with a response rate ranging from 19.9% to 92%. Only one study (20) determined the clinical picture of participants by a diagnostic interview by DSM criteria (12). The most part of the studies (17–19, 22, 23, 26–29) measured the level of psychological distress during or immediately after the outbreak. From 9.6% to 51% of HCWs reported symptoms of PTSD and from 20% to 75% reported the prevalence of anxiety and depression, respectively. The other studies (20, 21, 24, 30) rated psychological distress from one to three years after outbreak. PTSD symptoms were detected from 2% to 19% and from 5% to 90% reported psychiatric symptoms at follow-up. One study (38) reported in 19%–30% of HCWs significant levels of burnout. Only one study (19) comparing HCWs and healthy subjects did not report significant findings on the self-rating of perceived stress level. Only few studies compared the psychological burden of the outbreak comparing doctors and nurses: three did not find any differences (17, 26, 29), two reported a higher occurrence in nurses (28, 37) and the last one (22) a trend for nurses (**Table 1**).

**Table 2:** Summary of descriptive statistics of studies included (*N* = 15).

Variable	*N*	%
Total sample	7,766	
Doctors	577	7.4
Nurses	3,171	40.8
Other health care workers	1,306	16.8
Not specified	2,712	35.0
Singapore	3	20.0
Taiwan	2	13.3
Hong Kong	4	26.7
Canada	3	20.0
South Korea	1	6.7
Beijing	2	13.3
SARS	14	93.3
MERS	1	6.7
Cross-sectional and case-control	7	46.7
Cross-sectional	4	26.7
Case-control	4	26.7
General psychiatric symptoms	8	53.3
PTSD symptoms	10	66.6
Depression and anxiety symptoms	4	26.7
General psychological distress	4	26.7
Burnout	2	13.3
Mean of clinically relevant psychiatric symptoms	8	35.92 (19.17–52.67)
Mean of clinically relevant PTSD symptoms	8	17.24 (7.02–27.47)
Mean of clinically relevant depression and anxiety symptoms	2	6.4 (1.70–11.10)

Some studies (17, 20, 24, 26, 38) analyzed the buffering factors for the burden of outbreak on psychological distress. Protective factors were clear communication of directives/precautionary measures, support and training from supervisors/colleagues, years of health care experience and altruism; risk factors for depression were having been quarantined, high work exposure, current stressful job, high PTSD symptoms and pre-SARS trauma exposure.

Considering aggregated results, eight studies showed that up to 35% (95% CI: 19.17–52.67) of HCWs reported clinically significant levels of general psychiatric symptoms during and after pandemic emergency. Interestingly, pooled effect size (*d_w_* = .07 [−.11–.26]) did not highlight significant differences between HCWs who were and were not directly involved in addressing medical emergency. This evidence was consistent across studies included (*Q*_(2)_ = .16; *ns; I^2^ = *.00%). With respect to PTSD symptoms, the analyses found that 17% (95% CI: 7.02–27.47) of HCWs developed clinically significant symptoms of this conditions. Furthermore, the direct involvement in the management of pandemic emergency significantly affected the severity of PTSD symptoms (*d_w_* = .30 (.21–.39); *p* <.001), even though the heterogeneity across studies were large (*I^2^* = 72.05%) and significant (*Q*_(6)_ = 27.41; *p <*.01). Overall, clinically significant depressive and anxious symptoms were reported by up to 6% (95% CI: 7.02–27.47) of HCWs. Nevertheless, the HWCs who were directly involved in addressing pandemic emergency showed significantly higher depressive and anxious symptoms (*d_w_* = .66 (.46–.85); *p* <.001) than ones who were not directly exposed to the medical emergency. This finding was consistent across studies (*Q*_(2)_ = 2.93; *ns; I^2 =^* 31.78%).

Ultimately, Egger’s regression coefficients did not detect bias of publication for the previous indexes (**Table 3**). **Table 4** reported the rating of the risk of bias. Overall, the reviewed studies showed specific weaknesses in the participation rate, definition and measurement of exposure, and control of confounding variables. Anyhow, we must bear in mind that these real-world studies were performed in emergency contexts, and therefore their quality is acceptable though just sufficient from a methodological point of view.

**Table 3:** Pooled effect sizes concerning the effects of direct exposure to pandemic emergency.

Outcome	*N* direct exposure	*N* control subjects	*N* studies	*d_w_* (95%CI)	*Q* (df)	*I*^2^	Egger’s coefficient(95% bootstrap CI)
Overall psychiatric symptoms	271	761	3	.07 (−.11–.26)	.16 (2)	.00%	.58 (NE); *ns*
PTSD symptoms	624	1,948	7	.30 (.21–.39)***	27.41 (6)**	72.05%	1.56 (−25.28–10.39); *ns*
Depression and anxiety symptoms	638	1,571	3	.66 (.46–.85)***	2.93 (2)	31.78%	2.15 (NE); *ns*

**p <.01; ***p<.001; NE, not estimated.

**Table 4:** Assessment of risk of bias (*N* = 15).

Criteria	Yes	No	NA/NR
1. Was the research question or objective in this paper clearly stated?	14	0	1
2. Was the study population clearly specified and defined?	15	0	0
3. Was the participation rate of eligible persons at least 50%?	8	4	3
4. Were all the subjects selected or recruited from the same or similar populations (including the same time period)? Were inclusion and exclusion criteria for being in the study prespecified and applied uniformly to all participants?	15	0	0
5. Was a sample size justification, power description, or variance and effect estimates provided?	0	14	1
6. For the analyses in this paper, were the exposure(s) of interest measured prior to the outcome(s) being measured?	8	7	0
7. Was the timeframe sufficient so that one could reasonably expect to see an association between exposure and outcome if it existed?	15	0	0
8. For exposures that can vary in amount or level, did the study examine different levels of the exposure as related to the outcome (e.g., categories of exposure, or exposure measured as continuous variable)?	0	15	0
9. Were the exposure measures (independent variables) clearly defined, valid, reliable, and implemented consistently across all study participants?	5	10	0
10. Was the exposure(s) assessed more than once over time?	1	14	0
11. Were the outcome measures (dependent variables) clearly defined, valid, reliable, and implemented consistently across all study participants?	15	0	0
12. Were the outcome assessors blinded to the exposure status of participants?	1	0	14
13. Was loss to follow-up after baseline 20% or less?	0	1	14
14. Were key potential confounding variables measured and adjusted statistically for their impact on the relationship between exposure(s) and outcome(s)?	3	12	0
Total score	**100**	**77**	**33**

## Discussion

The COVID-19 pandemic presented as a significant challenge for healthcare services all over the world. The overload of healthcare systems for the burden of a new and unknown virus, the spread of diffusion, a significant lethality rate, and lack of definitive treatment protocols or vaccine represented some additional factors potentially influencing the psychological resources of HCWs.

Our findings evidence the likely link with mental problems of previous coronavirus outbreaks in terms of PTSD symptoms and other psychopathology (anxiety, depression, psychological distress) both in the acute phase and after a time interval in attenuated forms.

Unfortunately, almost all studies recruited convenience samples from well-defined, though small, populations reasonably due to this peculiar real-world research context. Beyond obvious problems of statistical power, sources of bias can be found in the insufficient measurement of the amount of exposure and in a poor evaluation of confounding variables (e.g. other sources of stress apart from working or not in high-risk settings, previous personal career, and so on). On the positive side, the reviewed studies highlight that evidence is not too dissimilar in various parts of the world, despite cultural and organizational differences. The most part of the studies adopted the Impact of Events Scale (IES) to detect PTDS symptoms, which have been diagnosed by a range of 20%–50% of health-care professionals. However, IES is a self-administered symptom scale to screen symptoms of PTSD. In addition, only one study (20) performed a vis-à-vis structured diagnostic interview and only 2% of subjects had a definite PTSD diagnosis after one year.

Possible psychopathological consequences of stress exposure include both specific sequelae (i.e. Adjustment Disorder, Acute Stress Disorder, Post-Traumatic Stress Disorder) and common mental disorders (e.g. Major Depressive Disorder, Generalized Anxiety Disorder, Substance-Related Disorders). Moreover, the emergence of a clinical condition among distressed individuals can be a new onset condition as well as a recurrence of previous disorders; finally, comorbid personality traits may play a role in the development of psychopathology among other predisposing factors (10). It is clear that a complete psychopathologic work-up should proceed with clinical interviews and psychometric tests, and self-administered tests on a voluntary basis may give only screening information. For this reason, we need studies assessing mental health of HCWs in a direct way, eventually adopting the cut-off of the screening tests to candidate people to the traditional procedure. Another critical point is the relevance of making follow-up study, because the cross-sectional design of most studies does not allow any prevision on the evolution of the clinical situation.

A rapid review on HCWs involved in COVID-19 pandemic (39) evidenced significant levels of distress, anxiety, depression and insomnia. Our study on previous coronavirus outbreaks adds a critical point, because we quantified the role of direct exposure to the risk of contagion (**Table 3**): if all HCWs showed a somewhat associated risk of developing psychiatric symptoms during outbreaks, only those in frontline showed a significant increased level of anxiety/depression and (then) PTSD. The wider study on HCWs involved in COVID-19 (40) had been performed in 34 hospitals of China and involved 1257 health care workers (68.7% response rate), with overall, 50.4%, 44.6%, 34.0%, and 71.5% of all participants reported symptoms of depression, anxiety, insomnia, and distress, respectively. The role of sleep disruption needs more studies, for the well-known link with psychopathology (41). Moreover, we need studies analyzing protective factors (both as institutional and personal ones) from the psychiatric outcome, to implement strategies of prevention.

A critical question is whether the health care workers who participated in these studies are representative of the entire population of HCWs. Unfortunately, the psychological mechanism motivating an individual to participate or not to a voluntary survey is unknown. Response bias may be present if the nonrespondents were either too stressed/depressed and/or anxious to respond or not at all stressed/depressed and/or anxious and therefore not interested in this survey.

Lancee and coworkers (20) evaluated new-onset episodes of psychiatric disorders in a mixed sample of 139 HCWs by using the Structured Clinical Interview for DSM-IV and the Clinician-Administered PTSD Scale, one to two years after the SARS-1 outbreak in Ontario. They found rates of lifetime prevalence for any mental disorder before the coronavirus pandemic, which were comparable to the Canadian community samples, including a lifetime prevalence rate of PTSD even lower than that of civilian samples in North America. Only a few new-onset episodes of common psychiatric disorders were detected (5%) including just one case of PTSD specifically attributable to the SARS experience. This small investigation was performed on subjects who were still in service on a voluntary basis (roughly one in four agreed to participate), and a critical question is whether the HCWs who participated in this study are representative of their colleagues; so, it is not informative under an epidemiological perspective.

The current COVID-19 outbreak might represent a matchless opportunity to study the burden and buffering factors of pandemic virus for mental health. This, in the perspective of planning an intervention for future epidemic outbreaks, both from the side of public health services and for the implementation of education strategies also focused on working in emergencies (e.g. core curriculum in clinical/emergency psychology in school of medicine and nursing). We do not know how many of HCWs participating to the survey have had a specific training on psychological issues, but we know that a lot of them have been called to manage difficult clinical decisions with strong ethical meanings, to communicate bad news, to remain quarantined from their families and kids, while maintaining overloading rhythms of work. In Lombardy (the most part of the Authors work in Lombardy, the Italian region with the worst situation related to COVID-19) (42), some of HCWs had to face the emergency without being allowed to choice if work or not in COVID wards (sometimes with different sub-specialty expertise as the case of dermatologists or neurologists called to work in intensive care). In many cases, there was not any psychological training to work in emergency. It is clear that each factor may have had a role in predicting the level of psychological burden of the medical emergency on HCWs, and these factors should be controlled in future research. Providing psychological support to frontline workers takes over as a significant public mental health challenge over the coming weeks and months (43). Some evidence exists that altruistic acceptance of the own role (24) and institutional support and training (38) may have a role in buffering the psychopathologic outcomes. However, we need more studies on resilience factors in HCWs. Given the adverse impacts of experiencing burnout, psychological distress in the workplace, it is of great importance to investigate the potential factors and mechanisms that could enlighten the improvement of the mental health and maintenance of adequate proficiency of HCWs in the midst of the pandemic. The role of the spouses and/or familial support, capacity of self-help and using mindfulness techniques to cope with distressing situations, personality characteristics, institutional facilities (e.g. mental health support, availability of medical supplies) deserve further studies. Moreover, we need to address factors bolstering resilience. Among all the influential factors, social support is one of the protective factors for mental health for HCWs (44–46). A strong social support network can buffer feelings of isolation, strengthening resilience. Video calls and virtual meetings (or on-line group support) allow for maintenance of social relations while preserving physical distancing.

Other moderating interventions include delivery of general and medical supplies, limiting isolation to the shortest duration necessary, and emphasizing altruism as core value of the profession as much as a strong leadership with clear, honest and open communication to balance fears and uncertainties (47).

Proposals for delivering psychological support exist (48), with better chance of achieving psychological interventions when clinical psychology units are available within the hospitals (a rarity in Italy). Telemedicine may be an opportunity for offering supportive interventions intended to promote wellness and boost coping strategy (such as empathic listening, psychoeducation or supportive therapy) (47).

In synthesis, our review showed an association with a likely negative burden for mental health of HCWs in terms of PTSD symptoms and other psychopathology (anxiety, depression, psychological distress) both in the acute phase and, in some cases, after a time interval. Learning lessons from the current pandemic outbreak is imperative to prepare better strategies for new healthcare management models for the next generations of doctors, nurses and staff of health-care services.

## Data Availability Statement

The raw data supporting the conclusions of this article will be made available by the authors, without undue reservation.

## Author Contributions

FG and RF ideated, wrote and called to collaborate the co-authors. GiP contributed to the theoretical building of the paper and wrote the parts on psychiatric issues, with a contribution in the selection of papers and quantification of the risk of bias. FR and FM made the bibliographic search. MC made the statistical analysis, giving some of the methodological indications. GaP, GS, MPC, AP supervised the all work, read and approved the manuscript. All authors contributed to the article and approved the submitted version.

## Conflict of Interest

The authors declare that the research was conducted in the absence of any commercial or financial relationships that could be construed as a potential conflict of interest.
